# Suppression of *pinoid* mutant phenotypes by mutations in *PIN-FORMED 1* and PIN1-GFP fusion

**DOI:** 10.1073/pnas.2312918120

**Published:** 2023-11-20

**Authors:** Michael Mudgett, Zhouxin Shen, Xinhua Dai, Steven P. Briggs, Yunde Zhao

**Affiliations:** ^a^Department of Cell and Developmental Biology, University of California San Diego, La Jolla, CA 92093-0116

**Keywords:** auxin, plant development, auxin transport, organogenesis, genetic interaction

## Abstract

Disruption of either the auxin transporter PIN-FORMED 1 (PIN1) or the protein kinase PINOID (PID) leads to the development of pin-like inflorescences. Previous studies have shown that phosphoregulation of PIN1 by AGC kinases including PID directs auxin flux to drive organ initiation. Here, we report unexpected findings on the genetic interactions between these two genes. We deleted the first 2/3 of the *PIN1* coding sequence using CRISPR/Cas9, and the resulting *pin1* mutant (*pin1-27*) was a strong allele. Surprisingly, heterozygous *pin1-27* suppressed two independent *pid* null mutants, whereas homozygous *pin1-27* enhanced the phenotypes of the *pid* mutants during embryogenesis. Furthermore, we show that deletion of either the hydrophilic loop or the second half of PIN1 also abolished PIN1 function, yet those heterozygous *pin1* mutants were also capable of rescuing *pid* nulls. Moreover, we inserted green fluorescent protein (GFP) into the hydrophilic loop of PIN1 through CRISPR-mediated homology-directed repair (HDR). The GFP signal and pattern in the *PIN1-GFP_HDR_* line are similar to those in the previously reported *PIN1-GFP* transgenic lines. Interestingly, the *PIN1-GFP_HDR_* line also rescued various *pid* null mutant alleles in a semidominant fashion. We conclude that decreasing the number of functional *PIN1* copies is sufficient to suppress the *pid* mutant phenotype, suggesting that PIN1 is likely part of a larger protein complex required for organogenesis.

## Significance

PIN auxin transporters and the PID (PINOID) kinase specify plant morphogenesis and organ formation by regulating dynamic gradients of the hormone auxin. Single *pin1* (PIN-FORMED 1) or *pid* mutants produce pin-like inflorescences without functional flowers while double mutants often lack cotyledons and do not grow past the seedling stage. Surprisingly, we found that *pid* mutants produced fertile flowers when a single copy of *PIN1* was mutated, suggesting that PID activity is obviated by reduced *PIN1* gene dosage. The finding of *PIN1* haplocomplementation of *pid* indicates that a multi-subunit complex which is sensitive to *PIN1* levels is essential for flower initiation. Further study into this complex using the genetic materials presented here will uncover the exact mechanisms by which auxin regulates floral organogenesis.

Loss-of-function mutations in *PIN-FORMED 1* (*PIN1*) lead to the development of pin-like inflorescences, phenocopying plants grown on media containing high concentrations of the polar auxin transport inhibitor, N-1-naphthylphthalamic acid (1, 2). PIN1 and its close homologs are auxin efflux carriers (3–6). PIN proteins are often polarly localized, enabling them to direct auxin flow and amplify auxin gradients, which are important for organogenesis and other key developmental processes (2, 7, 8). Genetic studies in *Arabidopsis* have identified several additional genes that, when mutated, fail to initiate flowers and phenotypically resemble *pin1*. When disrupted, PINOID (PID), a Ser/Thr protein kinase, causes the formation of pin-like phenotypes (9, 10). Disruption of Auxin Response Factor 5/MONOPTEROS, a transcription factor required for auxin signaling, also leads to the formation of pin-like inflorescences (11). Genetic screens for enhancers of *yuc1 yuc4* double mutants, which are defective in auxin biosynthesis, identified the *NAKED PINS IN YUC MUTANTS* (*NPY*) family of genes (12). The *yuc1 yuc4 npy1* triple mutants as well as the *npy1 npy3 npy5* triple mutants were phenotypically similar to *pin1* and *pid* (12, 13).

Beyond flower initiation, *PIN1* and *PID* have developmental roles during embryogenesis, as *pin1 pid* double mutants frequently fail to produce cotyledons (14). Interestingly, *pid* appears to provide a very sensitive background for identifying genes involved in the formation of cotyledons. The *NPY* family genes were previously identified as *pid* enhancers [called *enhancer of pinoid* (*enp*), or *macchi-bou 4* (*mab4*)] (15, 16). The *pid npy1/enp/mab4* double mutants completely lack cotyledons (12, 15, 16). Further inactivation of other auxin genes in the *pid* background leads to the same no-cotyledon phenotypes observed in *pid npy1* and *pid pin1*. For example, simultaneous inactivation of *PID* and its close homologs *PID2*, *WAG1*, and *WAG2* leads to the complete loss of cotyledons (13). Decreasing auxin biosynthesis in the *pid* background also prevents the formation of cotyledons. The *wei8 tar2* and the *yuc1 yuc4* double mutants, which are defective in the first and second steps of auxin biosynthesis, respectively, do not produce cotyledons in the *pid* background (17). Further genetic screens for *pid* enhancers identified additional genes important for cotyledon development. Mutations in *MOB1A*, a critical component in the Hippo signaling pathway, and *VPS28A*, a component of the endosomal sorting complex required for transport (ESCRT-I) complex, caused the failure of cotyledon development in the *pid* background (18–20).

Genetic studies of mutants that develop pin-like inflorescences or that fail to develop cotyledons have identified genes required for organogenesis in *Arabidopsis*, but how the genes are connected mechanistically is not fully understood. Studies have demonstrated that PID-mediated direct phosphorylation of PIN1 increases auxin efflux activity, strengthening PIN1-directed auxin flux (21). Additionally, PID kinase activity functions as a binary switch to shift PIN1 polarity from a basal to an apical orientation (22, 23). Thus, it is implied that *pid* mutant phenotypes are caused by mislocalization or inactivation of PIN1 (22, 23). PID was reported to directly phosphorylate specific sites of the PIN1 hydrophilic loop (23, 24). Several other kinases including D6 PROTEIN KINASE (D6PK) and Mitogen-Activated Protein Kinases have since been reported to alter PIN1 activity and polarity via phosphorylation (25–27). Interestingly, the conserved Ser/Thr residues in PIN proteins can be phosphorylated by both D6PK and PID kinases, and the PIN1 hydrophilic loop is still phosphorylated in *pid*, *wag1 wag2*, and *pid pid2 wag1 wag2* mutants (21, 28, 29). Nonetheless, D6PK and PID proteins appear to have divergent functions; ectopic expression of *D6PK* under the control of the *PID* promoter could not rescue *pid* phenotypes, and expression of *PID* using the *D6PK* promoter could not rescue *d6pk* mutants (21). These findings have led to the presumption that PID may modulate PIN1 polarity via a more complex mechanism than simply phosphorylating the PIN1 hydrophilic loop (29).

PIN proteins function as dimers and they are known to directly or indirectly interact with a variety of other proteins at the plasma membrane (4–6). The known PIN partners include the ATP-Binding Cassette B (ABCB) transporters such as ABCB1 and ABCB19, which stabilize PIN-containing auxin efflux complexes on the plasma membrane (30, 31). Furthermore, PID has been shown to interact with and phosphorylate TWISTED DWARF1, which is a regulator of ABCB auxin transporters, providing an indirect way for PID to regulate PIN-mediated auxin transport (32). PIN proteins physically interact with members of the NPY family and recruit them to the plasma membrane (33). Recently, SUE4, a PIN1-interacting membrane protein, was found to regulate the abundance of PIN1 (34). These findings indicate that PIN1 likely functions in a multiple-subunit complex during plant development.

Previous studies on the regulation of PIN1 by PID mainly relied on transgenic approaches and in vitro phosphorylation assays. Recent advances in CRISPR/Cas9 gene editing technologies enable generation of more precise modifications of *PIN1* and *PID*. In this study, we present our analyses of a series of insertion/deletion *pin1* mutants generated through CRISPR/Cas9 and characterize their interactions with various *pid* mutants. Surprisingly, a single copy loss-of-function mutation in *PIN1* was sufficient to rescue the fertility of *pid*, which is completely sterile, whereas homozygous *pin1* mutations enhanced the *pid* phenotypes during embryogenesis. Moreover, we show that *pid* phenotypes were suppressed by PIN1-GFP fusion, suggesting that the widely used PIN1-GFP fusion is not functionally equivalent to wild-type PIN1. These unexpected results reveal that *pid* mutants are sensitive to changes in *PIN1* gene dosage, suggesting that lower concentrations of PIN1 protein ameliorate a stoichiometric imbalance caused by *pid* knockout. This work establishes the existence of a PID-independent pathway for fertile flower generation and highlights the importance of characterizing the overall PIN1 complex.

## Results

### Generation of a New *pin1* Mutant Using CRISPR/Cas9.

**Fig. 1.**
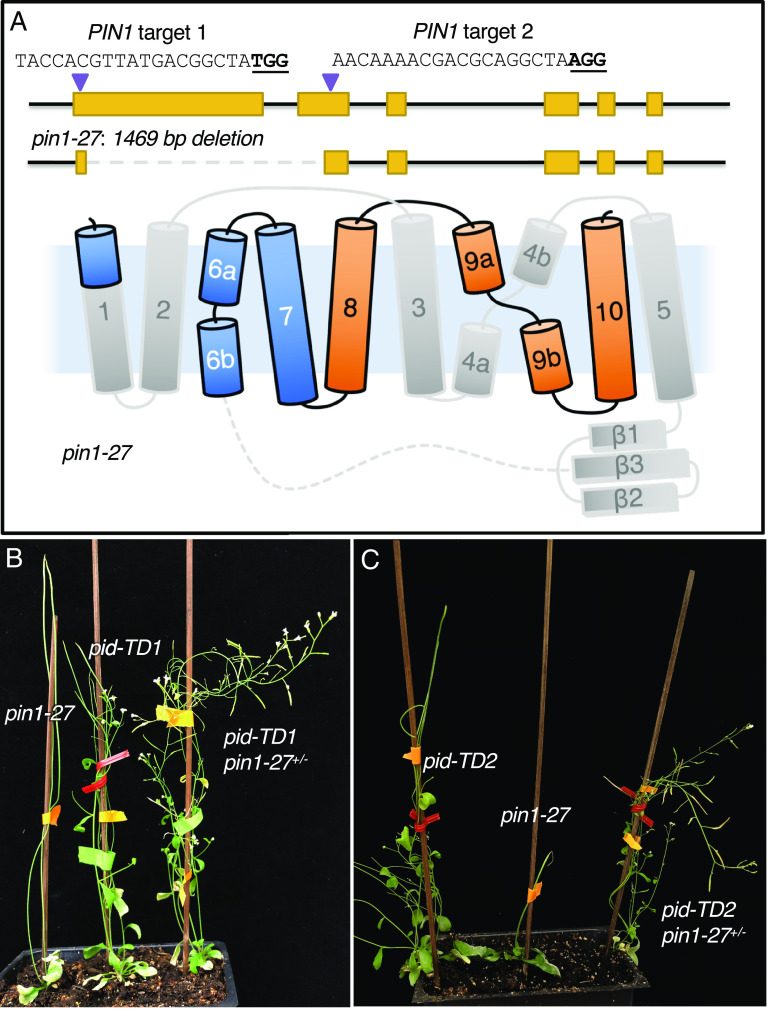
Suppression of *pid* T-DNA insertion mutants by a heterozygous loss-of-function *pin1* mutation. (*A*) Deletion of a large fragment of the *PIN1* gene using CRISPR/Cas9 gene editing technology. The first gRNA target is located 21 bp downstream of the *PIN1* start codon. The second target is in exon 2. The sequences of the two targets are shown, and the PAM sites are bolded and underlined. The *pin1-27* mutant contains a 1,469 bp deletion in *PIN1* that does not result in a frameshift. The deletion leads to a predicted PIN1 protein that lacks most of TMD 1, all of TMDs 2 to 5, and the entire hydrophilic loop. The deleted part of the PIN1 protein is marked in gray. (*B*) Suppression of a *pid* mutant by heterozygous *pin1-27.* Both *pin1-27* and *pid-TD1* produce pin-like inflorescences and are completely sterile. In the presence of one copy of *pin1-27*, *pid-TD1* produces many flowers and viable seeds. (*C*) Heterozygous *pin1-27* suppresses a second *pid* T-DNA insertion mutant.

We used two guide RNAs (gRNAs) to generate new loss-of-function *pin1* mutants (Fig. 1*A*). One gRNA was designed to cut soon after the start codon and the other cut near the end of exon 2; using them, we obtained a new mutant allele (*pin1-27*) that harbored a 1469 bp deletion in the *PIN1* gene (Fig. 1*A*) without a frameshift (*SI Appendix*, Fig. S1). Consequently, *pin1-27* was predicted to produce a mutated pin1 protein that is less than 1/3 of the wild-type PIN1 protein (183 amino acid residues vs. 622 amino acid residues) (*SI Appendix*, Fig. S1). The predicted pin1-27 protein lacks the entire hydrophilic loop, transmembrane domains (TMDs) 2, 3, 4, 5, and most of TMD 1 (Fig. 1*A*). The *pin1-27* allele was a strong allele and produced obvious pin-like inflorescences (Fig. 1 *B* and *C*). It had few leaves and the rare flowers it produced were sterile (Fig. 1 *B* and *C*). The phenotypes of *pin1-27* are similar to those from previously reported *pin1* mutants (2).

### Suppression of *pid* Null Mutants by Heterozygous *pin1-27*.

We crossed *pin1-27* to a *pid* mutant (*pid-TD1*) to study the genetic interactions between the two genes. The *pid-TD1* allele has a T-DNA insertion in exon 2 of *PID* (*SI Appendix*, Fig. S2) and has phenotypes similar to those of previously characterized strong alleles of *pid* mutants (Fig. 1*B*). Both *pin1-27* and *pid-TD1* made pin-like inflorescences and very few flowers (Fig. 1*B*). The *pin1-27 pid-TD1* double mutants completely abolished the development of cotyledons, a well-known phenotype reported in previous *pin1 pid* double mutants (14) (*SI Appendix*, Fig. S3*A*). Surprisingly, heterozygous *pin1-27* partially rescued the *pid-TD1* mutant (Fig. 1*B*), which is completely sterile on its own. Plants with a *pid-TD1 pin1-27^+/−^* genotype (^+/−^ refers to heterozygous for mutant and wild-type alleles) were able to develop many flowers and produced elongated siliques and viable seeds (Fig. 1*B*).

The suppression of the pin-like phenotypes of *pid-TD1* by heterozygous *pin1-27* was almost complete (Fig. 1*B*), but the suppression of the floral defects of *pid-TD1* was partial. It is known that *pid* mutants occasionally produce abnormal flowers with extra petals (*SI Appendix*, Fig. S3*B*). Heterozygous *pin1-27* was able to reduce the number and the size of *pid-TD1* petals (*SI Appendix*, Fig. S3 *B* and *C*). The reproductive organs of *pid-TD1* were often defective and lacked carpels and stamens (*SI Appendix*, Fig. S3*C*). In the *pin1-27^+/−^* background, *pid-TD1* regained the ability to make carpels and stamens (*SI Appendix*, Fig. S3*C*). However, the number of stamens was still fewer than that of wild type (WT). The carpel/gynoecium morphology of *pid-TD1 pin1-27^+/−^* was variable and quite different from that of WT (*SI Appendix*, Fig. S3*D*).

To determine whether suppression of *pid* by *pin1-27^+/−^* is allele specific, we crossed *pin1-27* to a second *pid* T-DNA mutant (*pid-TD2*), which also had a T-DNA insertion in the second exon (*SI Appendix*, Fig. S2). Like *pid-TD1*, *pid-TD2* was a strong allele; mutant plants developed pins and were sterile. The *pid-TD2 pin1-27* double mutants also failed to develop cotyledons (*SI Appendix*, Fig. S3*A*), but heterozygous *pin1-27* was able to restore the fertility of *pid-TD2* (Fig. 1*C*).

### The *pid-TD1* Mutant Is Suppressed by Several Heterozygous *pin1* Mutants.

**Fig. 2.**
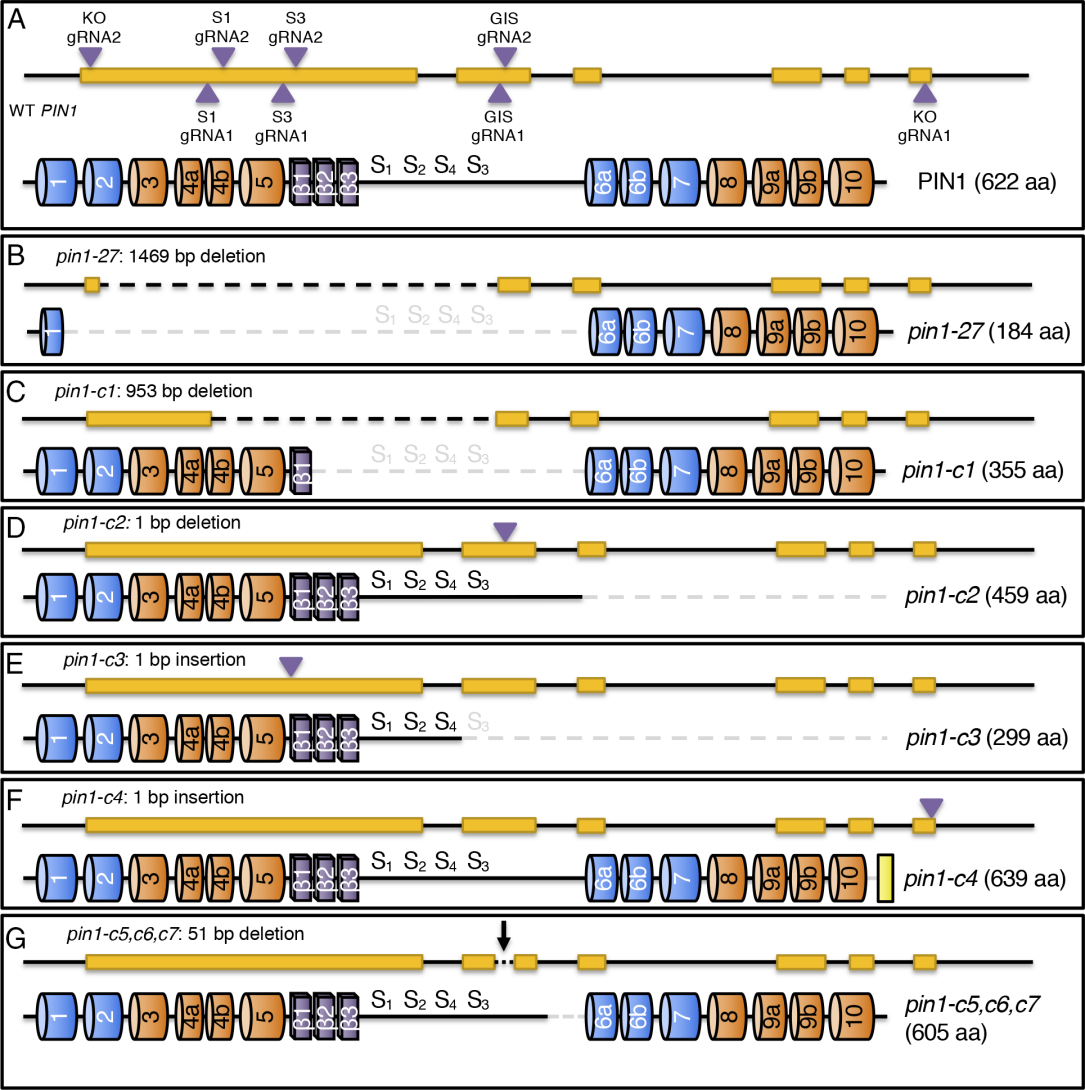
Generation of various mutations in *PIN1* using CRISPR/Cas9. (*A*) A schematic representation of the gRNA target sites in the *PIN1* gene and the domain structure of WT PIN1 protein. The exact target sequences are shown in *SI Appendix*, Table S1. (*B*–*F*) Molecular lesions of *pin1* mutants that can suppress *pid-TD1* when the *pin1* mutation is heterozygous. Large deletions are represented by a dotted line in both DNA and protein structures. The locations of small insertions or deletions are indicated by arrows. Note that *pin1-c4* contains an extra 21 residues, represented by a yellow box. (*G*) The *pin1* mutants that cannot suppress *pid-TD1.* Three independent *pin1* mutants that have the same 51 bp deletion in exon 2, which leads to a deletion of 17 amino acid residues, do not suppress the *pid-TD1* phenotype.

To rule out the possibility that a background mutation other than the *pin1-27* mutation accounts for the suppression of *pid* null mutants, we generated additional *pin1* mutants using CRISPR/Cas9 gene editing. As shown in Fig. 2*A*, we designed 8 gRNAs that targeted various sites in the *PIN1* gene. The target sequences are shown in *SI Appendix*, Table S1. Some of the gRNA combinations provided the chance to delete the conserved phosphorylation sites in PIN1 (Fig. 2*A*). The schematic structures of the WT *PIN1* gene and PIN1 protein along with their *pin1-27* counterparts are included in Fig. 2 *A* and *B* for comparison. We transformed the CRISPR plasmids into a population that was segregating wild-type *PID* with *pid-TD1* in the Columbia ecotype.

The first mutant (*pin1-c1*) we isolated contained a 953-bp deletion in the *PIN1* gene, which removed most of exons 1 and 2 (Fig. 2*C*) (*SI Appendix*, Fig. S4). The deletion maintained the open reading frame, indicating the potential production of a mutant pin1 protein that lacked the entire hydrophilic loop and two of the β sheets (Fig. 2*C*). The *pin1-c1* mutant was isolated in the *pid-TD1*^+/−^ background in the T1 generation, allowing us to analyze the phenotypes of *pin1-c1* and its interactions with *pid-TD1* in the T2 generation. Similar to the known *pid pin1* double mutants in the literature and those described above, *pin1-c1 pid-TD1* double homozygous mutants failed to develop cotyledons, suggesting that *pin1-c1* is a loss-of-function allele. Moreover, the *pin1-c1* single mutant produced pin-like inflorescences (*SI Appendix*, Fig. S5*A*), which were similar to those previously reported in *pin1* mutants. Interestingly, heterozygous *pin1-c1* was able to suppress *pid-TD1* (*SI Appendix*, Fig. S5*B*) to a similar extent as *pid-TD1 pin1-27^+/−^*(Fig. 1). Plants with the *pid-TD1 pin1-c1^+/−^*genotype were able to produce viable seeds (*SI Appendix*, Fig. S5*B*).

The deletion of an A in the second exon of *PIN1* caused a frameshift in *pin1-c2* (Fig. 2*D* and *SI Appendix*, Fig. S4), resulting in the complete removal of TMDs 6 to 10 from the PIN1 protein (Fig. 2*D*). Likewise, the insertion of a T in the first exon of *PIN1* led to a deletion of a large portion of the hydrophilic loop as well as TMDs 6 to 10 in *pin1-c3* (Fig. 2*E*). Both alleles were independently isolated in the *pid-TD1* homozygous mutant background in the T1 generation. In the T2 generation, all double homozygous *pid-TD1 pin1-c2* and *pid-TD1 pin1-c3* plants failed to develop cotyledons, while *pid-TD1 pin1-c2^+/−^* and *pid-TD1 pin1-c3^+/−^* plants were fertile (*SI Appendix*, Fig. S5 *C* and *D*).

The *pid* phenotypes were suppressed when a single copy of mutant *pin1* allele was present alongside wild-type PIN1. It is unlikely that the suppression was caused by a background mutation because each *pin1* mutant was generated independently and with different sets of gRNAs (Fig. 2). Furthermore, the above mutations result in predicted pin1 mutant proteins with significant truncations or gaps, which likely lead to complete loss of function (Fig. 2 *B*–*E*). Thus, our data suggest that *pid* suppression is triggered by the presence of a single *pin1* null allele, rather than the inclusion or deletion of a particular motif within *PIN1*. This claim is supported by the observation that the predicted protein sequences of *pin1-27* and *pin1-c3* are nearly mutually exclusive.

The *pin1-c4* allele was quite different from the *pin1-c* mutants described above (Fig. 2*F*). An A was inserted 11 bp upstream of the stop codon TGA in *pin1-c4* (Fig. 2*F* and *SI Appendix*, Fig. S4). The predicted *pin1-c4* protein only differed from WT PIN1 at the C-terminal region, replacing the WT C-terminal “LLGL” sequence with “HLGSMKRYYQNTGTLFYSFVG” in *pin1-c4* (Fig. 2*F*). The *pin1-c4* allele was isolated as *pin1-c4^+/−^ pid-TD1* in the T1 generation. At the T2 generation, all of the plants had cotyledons, suggesting that *pin1-c4* did not completely disrupt PIN1 function. At the young adult stage, *pid-TD1* phenotypes were enhanced by homozygous *pin1-c4*, as *pin1-c4 pid-TD1* double mutants made fewer leaves and displayed strong pin-like phenotypes (*SI Appendix*, Fig. S5*E*). Heterozygosity for *pin1-c4* also partially suppressed *pid-TD1* and *pin1-c4^+/−^ pid-TD1* plants produced viable offspring (*SI Appendix*, Fig. S5*F*).

### Not All *pin* Mutations Suppress *pid* Mutants.

When we transformed a *pid-TD1* segregating population with a construct that harbored the two gRNA units (GIS-gRNA1 and GIS-gRNA2) (Fig. 2*A*), we obtained multiple lines that had apparent deletions in the *PIN1* gene based on our PCR results. We analyzed three independent lines that were heterozygous for the *pid-TD1* locus at the T1 generation. At the T2 generation, none of the *pid-TD1* homozygous plants were rescued and we did not observe any plants that lacked cotyledons (*SI Appendix*, Fig. S5*G*). Sequencing results indicated that the three independent lines contained the same 51 bp deletion (Fig. 2*G*). The deletion led to a removal of 17 amino acid residues in the hydrophilic loop near TMD 6 which did not drastically alter the topology of the predicted protein product (Fig. 2*G*). Consequently, these three lines represent mutations in *PIN1* which do not appear to lead to a loss of function and thus have no obvious effect on *pid* phenotypes.

### Generation of *PIN1-GFP* Fusion Using CRISPR/Cas9-Mediated Homology-Directed Repair (HDR).

**Fig. 3.**
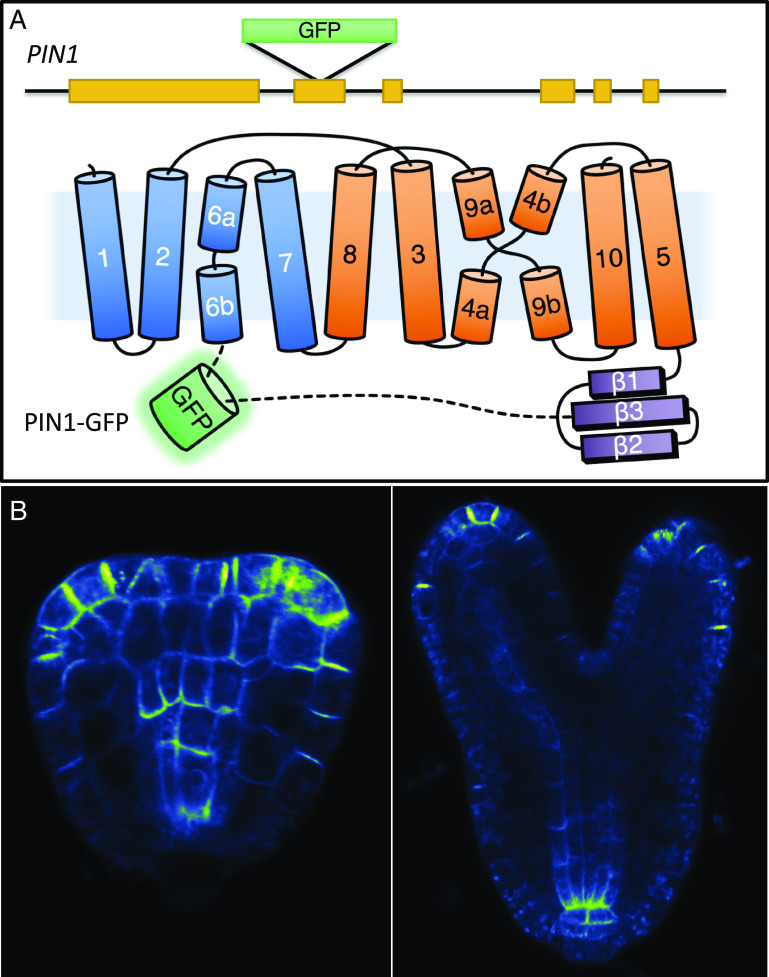
Generation of a PIN1-GFP fusion using CRISPR/Cas9-based HDR. (*A*) A schematic representation of the *PIN1* gene (*Top*) and the topology of the PIN1-GFP protein (*Bottom*). The *GFP* gene is inserted in-frame in the second exon of *PIN1*, resulting in a PIN1-GFP fusion. GFP is inserted between amino acid residues A452 and K453 near TMD 6. TMD helices 1, 2, 6, and 7 (marked in blue) are part of the scaffold domain. The other helices (marked orange) form the auxin transport domain. (*B*) The PIN1-GFP protein localizes to the plasma membrane with a pattern similar to that which has previously been reported.

It is known that adding a green fluorescent protein (GFP) tag at either the N terminus or C terminus of PIN1 disrupts PIN1 functions and that the fusions cannot complement *pin1* mutants. The most widely used *PIN1-GFP* lines have *GFP* inserted in the second exon of *PIN1* at the end of the hydrophilic loop, close to TMD 6 (Fig. 3*A*) (7). Previous *PIN1-GFP* lines were generated by transforming a plasmid that contains the preassembled *PIN1-GFP* unit under the control of the *PIN1* promoter (7).

Because expression of *PIN1-GFP* in transgenic plants is not stable in our laboratory conditions and the lines are difficult to genotype for zygosity, we generated a new *PIN1-GFP* line using CRISPR/Cas9-based HDR (35, 36). We inserted the *GFP* gene in the same location used in previously reported transgenic constructs (7). The *GFP* coding sequence without the stop codon was inserted seamlessly between amino acid residues A452 and K453 in the hydrophilic loop (Fig. 3*A*). The zygosity of our *PIN1-GFP HDR* line can be easily genotyped using a PCR-based method. We call the *PIN1-GFP* line we generated through CRISPR/Cas9-mediated HDR *PIN1-GFP_HDR_* to differentiate it from the previous *PIN1-GFP* transgenic lines.

The *PIN1-GFP_HDR_* homozygous line did not develop any pin-like inflorescences, consistent with previous studies that the reported insertion of *GFP* into the site did not abolish *PIN1* functions. The PIN1-GFP protein in the *PIN1-GFP_HDR_* line was visible under a microscope and displayed similar localization to that of transgenic *PIN1-GFP* lines (Fig. 3*B*).

### Suppression of *pid* Mutants by *PIN1-GFP_HDR_*.

**Fig. 4.**
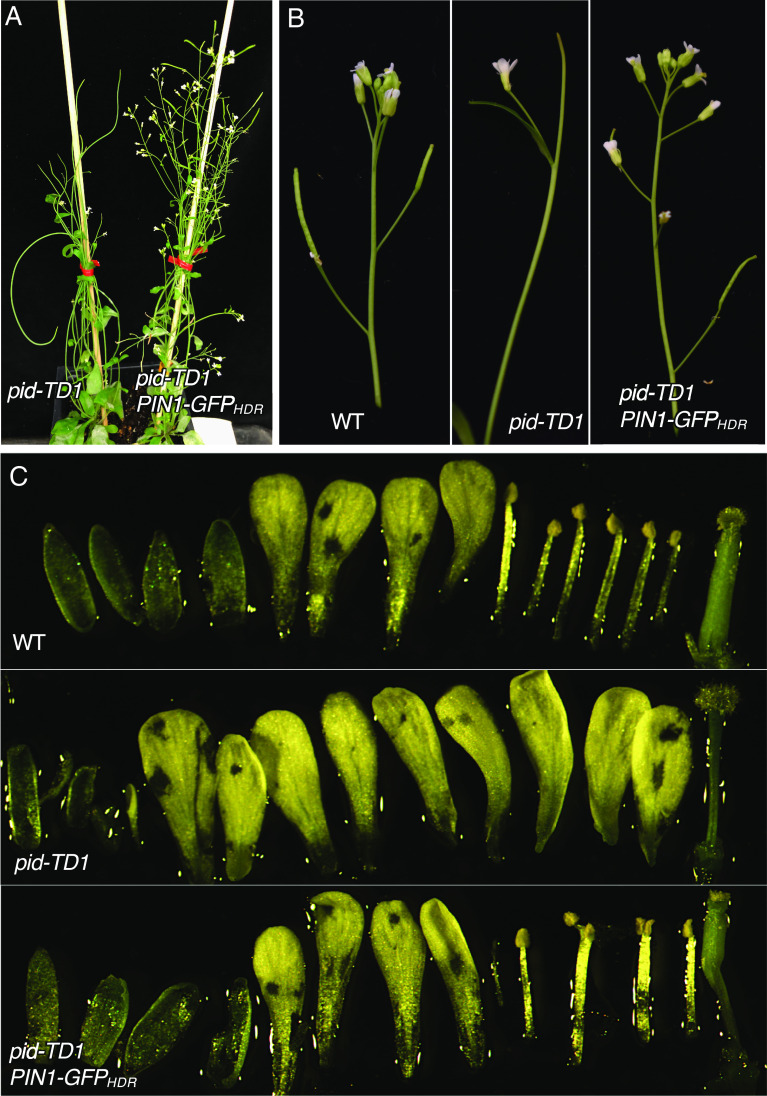
Partial suppression of a *pid* null mutant by PIN1-GFP_HDR_. (*A*) The sterile phenotype of *pid-TD1* is rescued by PIN1-GFP_HDR_ fusion. The *pid-TD1* plants make very few flowers, develop pin-like inflorescences, and are completely sterile (*Left*). In the *PIN1-GFP_HDR_* background, *pid-TD1* makes much more flowers and is fertile (*Right*). (*B*) Comparison of the inflorescence apex of WT, *pid-TD1*, and *pid-TD1/ PIN1-GFP_HDR_*. Note that *pid-TD1 PIN1-GFP_HDR_* produces elongated siliques and does not form a pin-like inflorescence. (*C*) Floral defects of *pid-TD1* are only partially rescued. WT flowers usually have four sepals, four petals, six stamens, and two fused carpels (*Top*). Flowers from *pid-TD1* plants have multiple petals, often lack stamens, and have gynoecia without valves (*Middle*). Flowers in *pid-TD1 PIN1-GFP_HDR_* develop functional stamens and carpels and fewer petals than *pid-TD1*. However, some stamens are fused and carpels are noticeably shorter than those of WT (*Bottom*).

We crossed the *PIN1-GFP_HDR_* line to *pid-TD1* to study whether PIN1-GFP localization and polarity would be affected by the absence of the PID protein. To our surprise, the phenotypes of homozygous *pid-TD1* were rescued by homozygous *PIN1-GFP_HDR_* (Fig. 4*A*). Plants with the double homozygous *pid-TD1 PIN1-GFP_HDR_* genotype were fertile and hardly produced any pin-like inflorescences (Fig. 4*B*). Flowers of *pid-TD1 PIN1-GFP_HDR_* plants had fewer petals relative to *pid-TD1* plants (Fig. 4*C*). Relative to *PIN1-GFP_HDR_* plants, flowers from *pid-TD1 PIN1-GFP_HDR_* plants had similar numbers of petals, but fewer stamens and abnormal gynoecia (Fig. 4*C*). Suppression of homozygous *pid-TD1* by *PIN1-GFP_HDR_* appeared semidominant, as plants heterozygous for *PIN1-GFP_HDR_* were able to produce both pin-like inflorescences and fertile flowers in the *pid-TD1* background (*SI Appendix*, Fig. S6).

### Suppression of *pid* by *PIN1-GFP_HDR_* Is Not Allele Specific.

We generated two new *pid* deletion mutants using CRISPR/Cas9 technology (*SI Appendix*, Fig. S7). The first, *pid-c1*, lacked almost the entire *pid* coding region including the start codon (*SI Appendix*, Fig. S7). The second CRISPR mutant allele, *pid-c2*, contained a 13 bp deletion in the second exon (*SI Appendix*, Fig. S7). Both *pid-c1* and *pid-c2* were strong alleles and produced pin-like inflorescences. The two CRISPR *pid* mutants were completely sterile. In the presence of *PIN1-GFP_HDR_*, both *pid-c1* and *pid-c2* were suppressed in a semidominant manner (*SI Appendix*, Fig. S7*B*).

### Phosphorylation of *PIN1-GFP* Fusion and *PIN* Proteins Does Not Require PID.

**Fig. 5.**
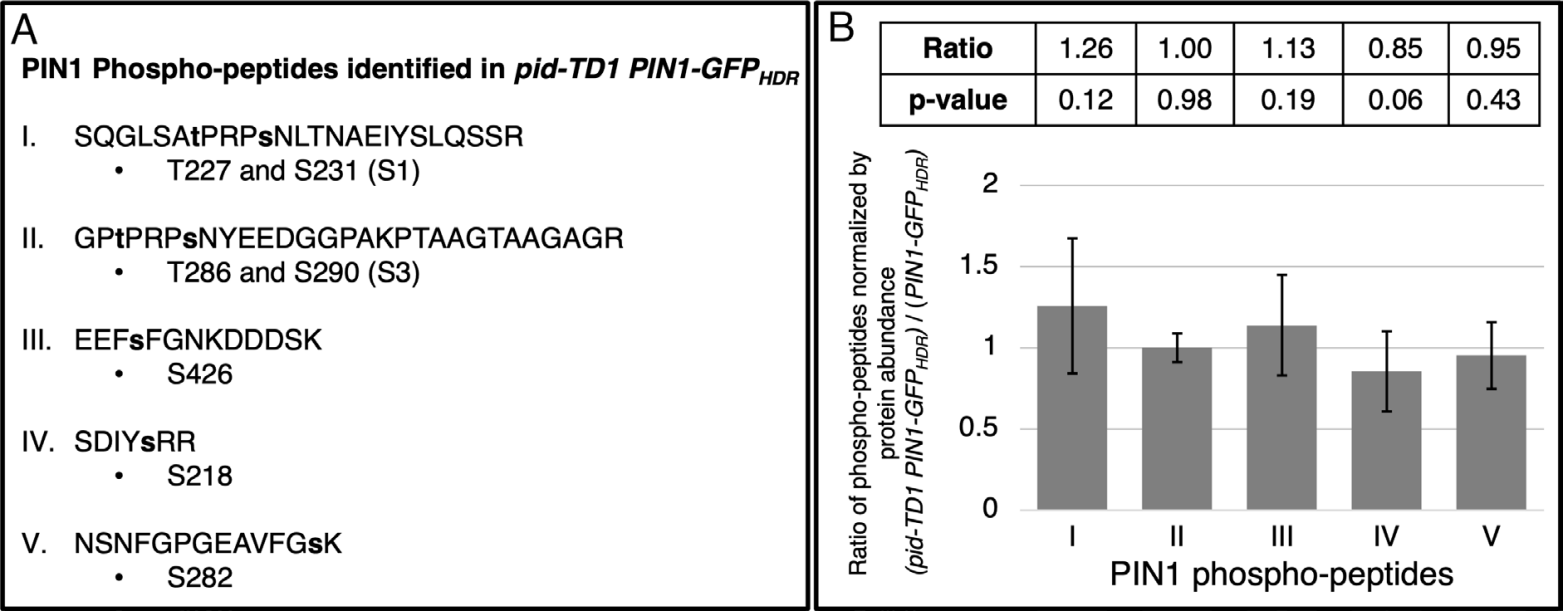
Phospho-proteomic analysis of PIN1. (*A*) Five PIN1 phospho-peptides, which contain seven phosphorylated Serine and Threonine residues, were identified in *pid-TD1 PIN1-GFP_HDR_* tissue. The phosphorylated residues are shown in lowercase and bolded. (*B*) Ratios of the identified phospho-peptides in the *pid-TD1 PIN1-GFP_HDR_* background compared to *PIN1-GFP_HDR_*.

Phosphorylation/dephosphorylation regulates the polarity and activity of PIN proteins (28, 37). Many Ser/Thr residues in the hydrophilic loop of PIN proteins have been identified as conserved kinase targets (*SI Appendix*, Fig. S8) (28). Among them, the serine residues S1 to S4 have been suggested as PID targets (*SI Appendix*, Fig. S8). The availability of homozygous *pid* null seeds in the *PIN1-GFP_HDR_* background enabled us to analyze the phosphorylation status of PIN proteins in the absence of PID. We conducted phospho-proteomic analysis of WT, the *PIN1-GFP_HDR_* line, and *pid-TD1 PIN1-GFP_HDR_* plants (Dataset S1). We observed five phospho-peptides derived from PIN1 in WT plants (Fig. 5). The same five phospho-peptides were also observed in both *PIN1-GFP_HDR_* plants and *pid-TD1 PIN1-GFP_HDR_* plants. The previously characterized phosphorylation sites in PIN1, S1 and S3, were phosphorylated in the presence and absence of *PID* (Fig. 5*A* and *SI Appendix*, Fig. S8). Likewise, the abundances of phospho-PIN1 peptides were not reduced by the removal of the PID kinase (Fig. 5*B*). Additionally, we noticed that phosphorylation of the S3 site in PIN3, PIN4, and PIN7 was also not reduced in the *pid* mutant (Dataset S1), indicating that PID is not required for the phosphorylation of this particular serine residue in additional protein family members. These results confirm and extend previously reported findings that PIN1 is phosphorylated in *pid, wag1 wag2,* and *pid pid2 wag1 wag2* mutants (21, 29).

## Discussion

In this paper, we presented the unexpected phenotypic suppression of *pid* null mutants by heterozygous loss-of-function *pin1* mutants and PIN1-GFP_HDR_ fusion. It has previously been shown that single loss-of-function *pin1* mutants and single loss-of-function *pid* mutants develop similar pin-like inflorescences, suggesting that *PID* and *PIN1* participate in the same pathway. Likewise, the enhanced phenotype (defective cotyledon development) in *pin1 pid* double mutants indicates a clear genetic interaction between the two. These observations, combined with the evidence that PID can phosphorylate conserved sites in PIN1, lend themselves to a model where PID directly phosphorylates PIN1 in order to regulate PIN1 polarity and/or activity and positively influence flower development (22, 28, 29). Because the evidence suggests that both PID and PIN1 are uniquely required for fertile flower initiation, it was surprising to observe that heterozygous *pin1* mutants could suppress *pid* phenotypes.

Interestingly, although *pin1* and *pid* mutants share the pin-like inflorescence phenotype, they differ in other aspects of development. Notably, mutations in *PIN1* and *PID* lead to opposite effects on leaf development. The *pin1* mutants generally have fused or single cotyledons and fewer true leaves than WT whereas *pid* mutants often have three cotyledons and more true leaves, suggesting that PIN1 and PID have different roles depending on the developmental context of their expression (38, 39). One hypothesis that can account for the observed phenotypic differences between *pin1* and *pid* is that PID regulates interactions between PIN1 and its partners by phosphorylating PIN1 and/or PIN1 partners. Specific PIN1 partners may only be present in particular developmental contexts.

Although the exact molecular mechanism responsible for the observed phenotypic suppression of *pid* null mutants by heterozygous *pin1* mutations and PIN1-GFP_HDR_ is still not clear, the results presented in this paper are consistent with a hypothesis wherein flower formation depends on the correct dosage of active PIN1 protein, which affects the stoichiometry of PIN1 and its partners. PIN proteins are known to form complexes with other proteins including NPY family proteins, ABCB transporters, and SUE4 (30, 31, 33, 34). PID is also known to regulate ABCBs through TWD1 (32) and *pid* mutants synergistically interact with *npy* mutants (12, 15, 16). In the absence of PID, the stoichiometry of PIN1 relative to its partners is off-balance, leading to defects in flower formation. Mutating a single copy of *PIN1* lowers the gene dosage of *PIN1*, which could have a correcting effect on the stoichiometric imbalance between PIN1 and its partners. Our observation of a roughly 50% decrease in PIN1 protein levels in *pin1-27^+/−^ pid-TD2* and *pin1-c2^+/−^ pid-TD1* plants is consistent with this model (*SI Appendix*, Fig. S9).

This hypothesis relies on the assumption that the activity or stability of PIN1-GFP_HDR_ is lower than that of wild-type PIN1 such that the effective PIN1 dose is lower in *PIN1-GFP_HDR_* plants, which we have not demonstrated here. Regardless, the PIN1-complex hypothesis would be bolstered by additional studies investigating plants with tunable PIN1 levels by overexpression, promoter mutagenesis, or RNAi-induced knockdown. Furthermore, isolation of a *pin1* allele where the coding sequence has been completely eliminated will determine whether the hypothesized partial pin1 mutant proteins presented in this study are acting only as loss-of-function alleles or are impacting the wild-type PIN1 proteins independently of PID.

Haploinsufficiency has been well documented in yeast, animals, and plants, but usually, the homozygous and heterozygous mutants have similar phenotypes with different severity (40). The results presented here describe a unique relationship wherein *pin1* and *pid* share the single mutant pin-like inflorescence phenotype, they have an enhanced double-mutant phenotype, and *PIN1* heterozygosity has a restorative effect on *pid*. We would like to call the unique genetic interaction between *pid* and *pin1* haplocomplementation (HC), which contrasts with complementation, haploinsufficiency, and haplosufficiency. HC occurs when a gene can provide its function by expressing only 50% of its dose in the presence of full loss of function of a second gene in the same pathway, whereas 0% or 100% of the first gene fails to suppress the mutation in the second gene.

Overall, our results reveal that the contributions of PIN1 and PID to flower formation are not simply linear or additive. The various new *pin1* and *pid* mutants and the stable *PIN1-GFP_HDR_* line presented in this work provide essential tools for future experimentation. The availability of homozygous fertile *pid* null materials enables genetic dissection of the pathway in which both PID and PIN1 are key components.

## Materials and Methods

Mutants and WT plants used in this study are the *Arabidopsis* Columbia ecotype. CRISPR knockout mutants were generated using the modified *pHEE401* vector, which uses an egg-cell specific promoter to drive *Cas9* expression and the *Arabidopsis U6-26* and *U6-29* promoters to control gRNA production (41). We added an mCherry unit (42) to the *pHEE401* vector so that transgenics and nontransgenics can be easily differentiated. gRNA targets used for generating mutations in *PIN1* and *PID* are listed in *SI Appendix*, Table S1. Genotyping primers are listed in *SI Appendix*, Table S2.

The *PIN1-GFP HDR* line was generated using the sequential transformation method previously reported (35). The *DD45-Cas9* transgenic line generated by Jian-kang Zhu (35) was obtained from the ABRC (https://abrc.osu.edu/). The *PIN1-GFP HDR* line was genotyped using the PCR primers listed in *SI Appendix*, Table S2.

### Proteomics Method.

About 0.5 g of frozen flower tissue was ground in liquid nitrogen by a mortar and pestle for 15 min into fine powder and then transferred to a 50-mL conical tube. Proteins were precipitated and washed by 50 mL of −20 °C acetone three times and then by 50 mL of −20 °C methanol three times. Samples were centrifuged at 4,000 × g for 10 min at 4 °C. The supernatant was removed and discarded.

Protein pellets were suspended in extraction buffer [8M Urea/100 mM Tris/10 mM N-ethylmaleimide/phosphatase inhibitors, pH 7]. Proteins were first digested with Lys-C (Wako Chemicals, 125-05061) at 37 °C for 15 min. Protein solution was diluted 8 times to 1M urea with 100 mM Tris and digested with trypsin (Roche, 03708969001) for 12 h.

Digested peptides were purified on a Waters Sep-Pak C18 cartridge, eluted with 60% acetonitrile. TMT-18 labeling was performed in 60% acetonitrile/100 mM Hepes, pH 7. TMT labeling efficiency was checked by LC-MS/MS to be greater than 99%. Labeled peptides from different samples were pooled together. Cysteines were reduced by 10 mM TCEP and alkylated by adding 20 mM N-methylmaleimide and incubating at 37 °C for 30 min. 150 μg of pooled peptides was analyzed by 2D-nanoLC-MS/MS for total proteome profiling, and 1 mg of total peptides was used for phosphopeptide enrichment.

Phosphopeptide enrichment was performed using CeO_2_ affinity capture. 20% colloidal CeO_2_ (Sigma, 289744) was added to the acidified peptide solution (1 mg peptide in 1 mL 1% TFA/2M lactic acid/60% acetonitrile, CeO_2_:peptide w:w ratio = 4:1). After brief vortexing, CeO_2_ with captured phosphopeptides was spun down at 5,000 g for 1 min. The supernatant was removed, and the CeO_2_ pellet was washed with 1 mL of 1% TFA/2M lactic acid/60% acetonitrile. Phosphopeptides were eluted by adding 200 μL eluting buffer (200 mM (NH_4_)_2_HPO_4_, 2M NH_3_.H_2_O, and 10 mM EDTA, pH 9.5) and vortexing briefly. CeO_2_ was precipitated by adding 40 μL 2M citric acid to a final pH of 3. The sample was centrifuged at 16,100 g for 1 min. The supernatant containing phosphopeptides was removed and ready for mass spectrometry analysis.

An Agilent 1100 HPLC system was used to deliver a flow rate of 600 nL/min to a custom 3-phase capillary chromatography column through a splitter. Column phases were a 20-cm long reverse phase (RP1, 5 μm Zorbax SB-C18, Agilent), 6-cm-long strong cation exchange (SCX, 3 μm PolySulfoethyl, PolyLC), and 20-cm-long reverse phase 2 (RP2, 3.5 μm BEH C18, Waters), with the electrospray tip of the fused silica tubing pulled to a sharp tip (inner diameter <1 μm). Peptide mixtures were loaded onto RP1, and the 3 sections were joined and mounted on a custom electrospray adapter for on-line nested elutions. Peptides were eluted from RP1 to SCX using a 0 to 80% acetonitrile gradient for 60 min and then were fractionated by the SCX column section using a series of 20 step salt gradients of ammonium acetate over 20 min, followed by high-resolution reverse phase separation on the RP2 section of the column using an acetonitrile gradient of 0 to 80% for 210 min.

Mass spectra were acquired on a Q Exactive HF mass spectrometer (Thermo Electron Corporation, San Jose, CA) operated in positive ion mode with a source temperature of 275 °C and spray voltage of 3 kV. Automated data-dependent acquisition was employed on the top 20 ions with an isolation window of 1.2 Da and collision energy of 30. The mass resolution was set at 60,000 for MS and 30,000 for MS/MS scans, respectively. Dynamic exclusion was used to improve the duty cycle.

The raw data were extracted and searched using Spectrum Mill vBI.07 (Broad Institute of MIT and Harvard). MS/MS spectra with a sequence tag length of 1 or less were considered to be poor spectra and were discarded. The remaining high-quality MS/MS spectra were searched against the *Arabidopsis* TAIR10 protein database. A 1:1 concatenated forward-reverse database was constructed to calculate the false discovery rate (FDR). Common contaminants such as trypsin and keratin were included in the protein database. There were 70,802 protein sequences in the final protein database. Search parameters were set to Spectrum Mill’s default settings with the enzyme parameter limited to full tryptic peptides with a maximum mis-cleavage of 1. Cutoff scores were dynamically assigned to each dataset to obtain the FDRs of 0.1% for peptides and 1% for proteins. Phosphorylation sites were localized to a particular amino acid within a peptide using the variable modification localization score in the Spectrum Mill software. Proteins that share common peptides were grouped using principles of parsimony to address protein database redundancy. Total TMT-18 reporter intensities were used for relative protein quantitation. Peptides shared among different protein groups were removed before TMT quantitation. Isotope impurities of TMT-18 reagents were corrected using correction factors provided by the manufacturer (Thermo). Median normalization was performed to normalize the protein TMT-18 reporter intensities in which the log ratios between different TMT-18 tags were adjusted globally such that the median log ratio was zero.

## Supplementary Material

Appendix 01 (PDF)

Dataset S01 (XLSX)

## Data Availability

The raw spectra for the proteome data have been deposited in the Mass Spectrometry Interactive Virtual Environment (MassIVE) repository (massive.ucsd.edu/ProteoSAFe/static/massive.jsp, accession ID MSV000092373) (43). All other data are included in the manuscript and/or supporting information.
