# Supplementary material for: Suppression of pinoid mutant phenotypes by mutations in PIN-FORMED 1 and PIN1-GFP fusion
Source: Proc Natl Acad Sci U S A. 2023 Nov 20;120(48):e2312918120. doi: 10.1073/pnas.2312918120 (PMC10691239; doi:10.1073/pnas.2312918120)
Supplement: Supplementary file 1 — Appendix 01 (PDF) [file pnas.2312918120.sapp.pdf]

PNAS

Supporting Information for
Suppression of pinoid (pid) mutant phenotypes by mutations in PIN-FORMED1 (PIN1) and PIN1-GFP fusion

Michael Mudgett, Zhouxin Shen, Xinhua Dai, Steven P. Briggs*, and Yunde Zhao*

Department of Cell and Developmental Biology, University of California San Diego, 9500 Gilman Drive, La Jolla, CA 92093-0116

For Correspondence: yundezhao@ucsd.edu or sbriggs@ucsd.edu

This PDF file includes:

Figures S1 to S9
Tables S1 to S2

Other supporting materials for this manuscript include the following:

Dataset S1

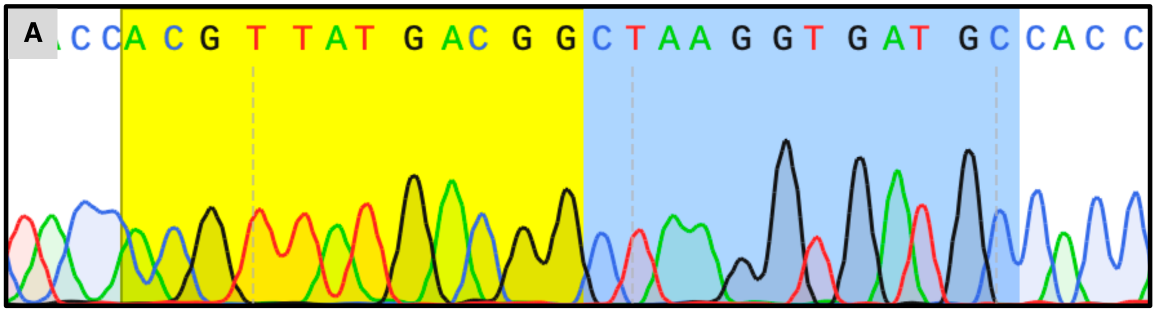

B *pin1-27* cDNA:

ATGATTACGGCGGCGGACTTCTACC**ACGTTATGACGG**CTAAGGTGATGCCACCAACAAGTGTGATG
ACAAGACTCATTCTCATTATGGTTTGGAGGAAACTTATTCGTAATCCCAACTCTTACTCCAGTTTA
TTCGGCATCACCTGGTCCCTCATTTCCTTCAAGTGGAACATTGAAATGCCAGCTCTTATAGCAAAG
TCTATCTCCATACTCTCAGATGCAGGTCTAGGCATGGCTATGTTCAGTCTTGGGTTGTTCATGGCG
TTAAACCCAAGAATAATAGCTTGTGGAAACAGAAGAGCAGCTTTTGCGGCGGCTATGAGATTTGTC
GTTGGACCTGCCGTCATGCTCGTTGCTTCTTATGCCGTTGGCCTCCGTGGCGTCCTCCTCCATGTT
GCCATTATCCAGGCAGCTTTGCCGCAAGGAATAGTACCGTTTGTGTTTGCCAAAGAGTATAATGTG
CATCCTGACATTCTTAGCACTGCGGTGATATTTGGGATGTTGATCGCGTTGCCCATAACTCTTCTC
TACTACATTCTCTTGGGTCTATGA

C pin1-27 protein:

MITAADFY**HVMT**AKVMPPTSVMTRLILIMVWRKLIRNPNSYSSLFGITWSLISFKWNIEMPALIA
KSISILSDAGLGMAMFSLGLFMALNPRIIACGNRRAAFAAAMRFVVGPAVMLVASYAVGLRGVLL
HVAIIQAALPQGIVPFVFAKEYNVHPDILSTAVIFGMLIALPITLLYYILLGL

Fig. S1. Generation of a large deletion in the *PIN1* gene. A) Sanger sequencing of *pin1-27*. The deletion to form *pin1-27* occurred between the highlighted traces. The *pin1-27* allele produces a smaller cDNA (B) and a smaller protein than WT (C). The deletion occurred between the bolded and underlined sequence.

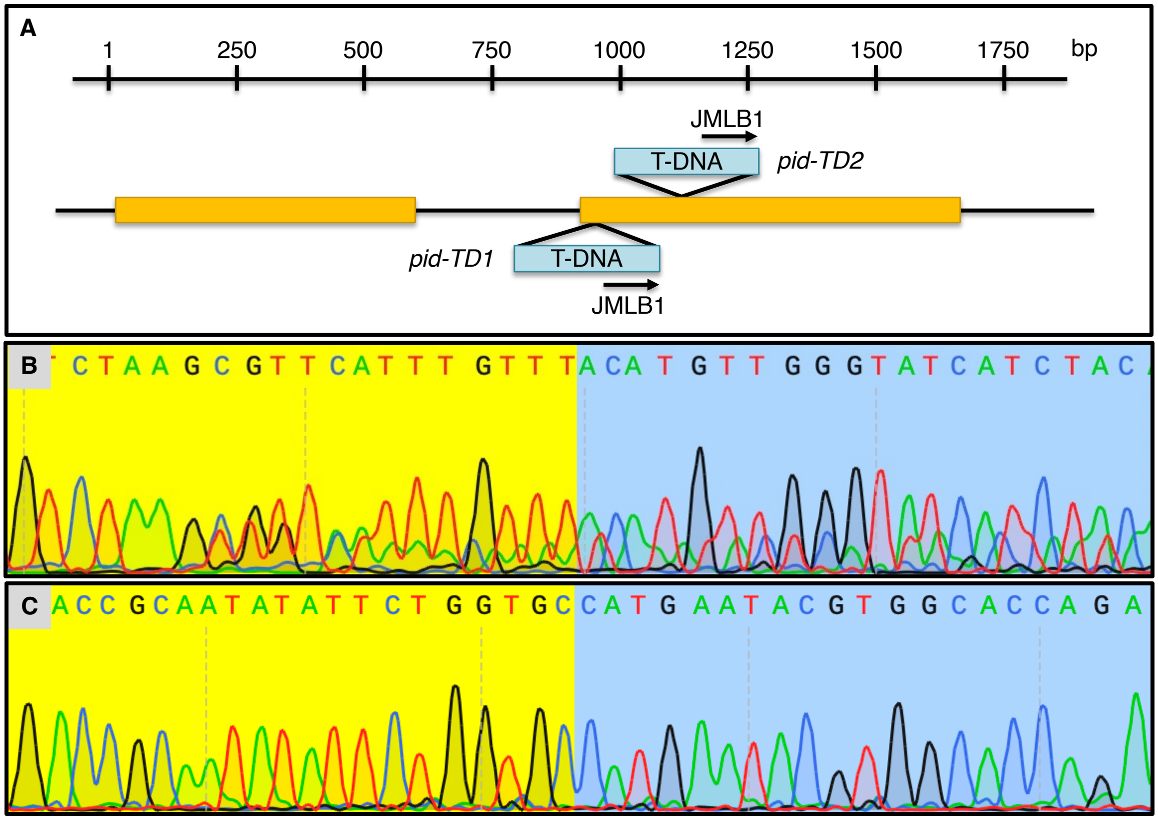

Fig. S2. Sequence analysis of the two T-DNA insertion *pid* mutants used in this study. A) A schematic representation of the T-DNA insertion sites of *pid-TD1* and *pid-TD2*. The junction was sequenced using the T-DNA primer JMLB1 (5′-GGCAATCAGCTGTTGCCCGTCTCACTGGTG-3′). B) Sequencing trace of *pid-TD1*, in which the T-DNA was inserted at 896 bp downstream of the *PID* start codon. C) The *pid-TD2* allele has a T-DNA insertion at 1165 bp downstream of the *PID* start codon. T-DNA and other sequences are highlighted in yellow and genomic *PID* sequence is highlighted in blue.

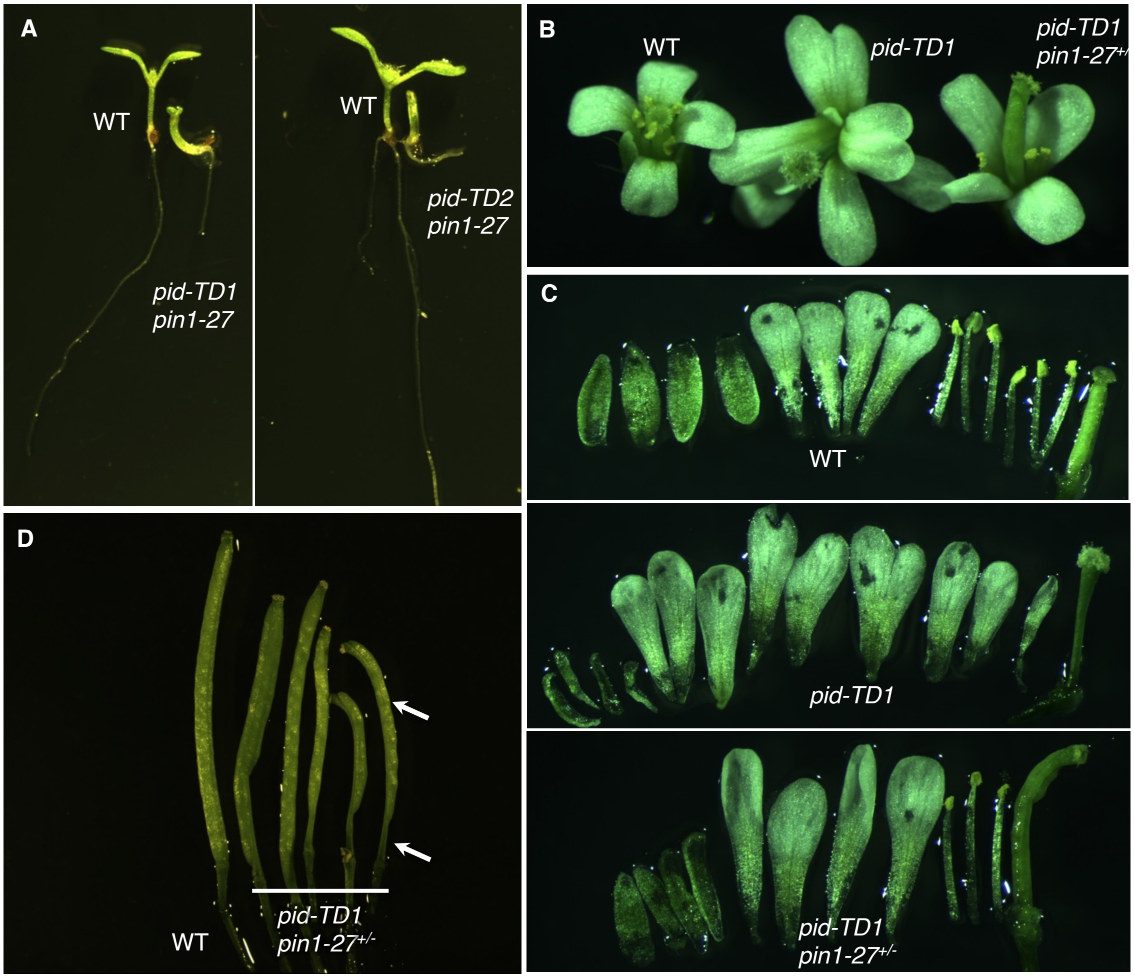

Fig. S3. Partial suppression of *pid* T-DNA mutants by heterozygous *pin1-27*. A) The *pid pin1-27* double mutants lack cotyledons. B) Suppression of the floral defects of *pid-TD1*, which occasionally produces abnormal flowers. Heterozygosity of *pin1-27* reduces the number and size of the petals in *pid-TD1*. C) Comparison of floral organs in WT (top), *pid-TD1* (middle), and *pid-TD1 pin1-27*$^{+/-}$ (bottom). WT flowers usually have four sepals, four petals, six stamens, and two carpels fused as a gynoecium. The *pid-TD1* mutant flower produces more petals which are occasionally fused. Flowers of *pid-TD1* often lack stamens and have thin gynoecia without viable ovules. Flowers of *pid-TD1* in the heterozygous *pin1-27* background have fertile stamens and carpels. The color in these photos is greener due to the lighting system used. D) The siliques of *pid-TD1 pin1-27*$^{+/-}$ vary in morphology and produce viable seeds. The siliques from *pid-TD1 pin1-27*$^{+/-}$ plants often have longer gynophores and just one valve (arrows).

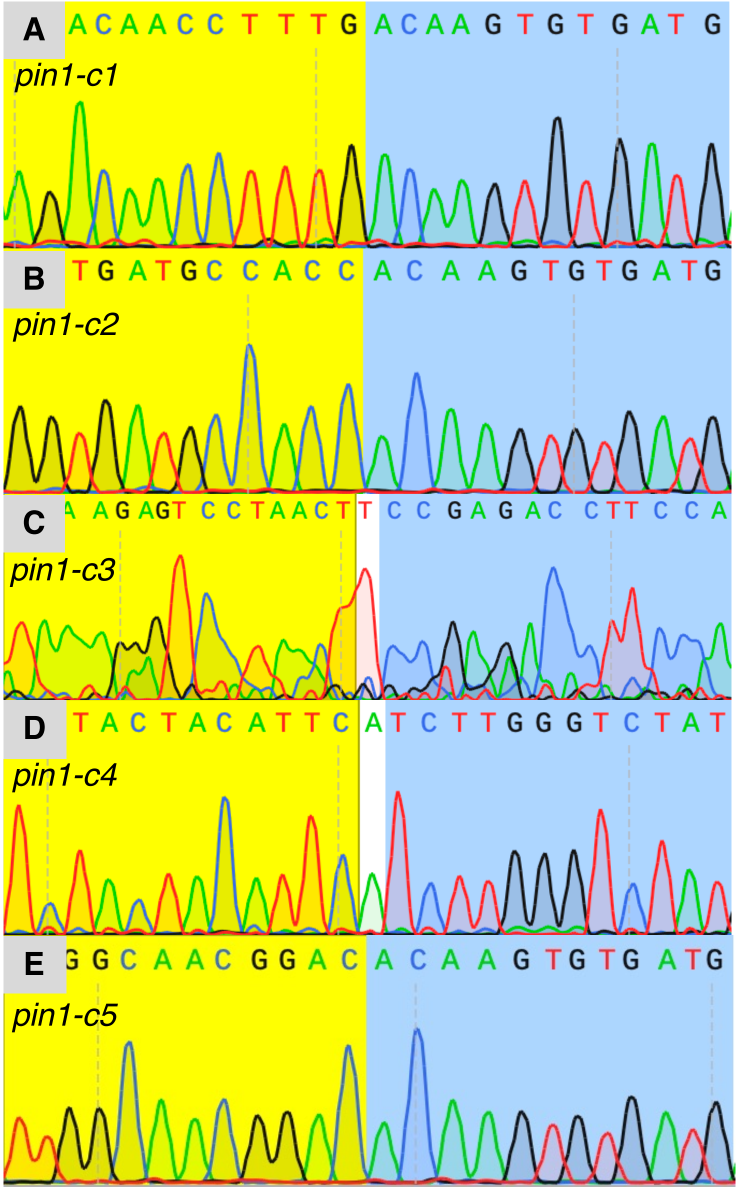

Fig. S4. DNA sequencing results of the new *pin1* mutations generated by CRISPR/Cas9. A) *pin1-c1.* A 953 bp fragment in the *PIN1* gene is deleted in *pin1-c1*. The deletion did not cause a frameshift. B) *pin1-c2.* Deletion of a single A, 1523 bp downstream of the *PIN1* start codon, caused a frameshift and the deletion of the C-terminal half of the PIN1 protein. C) *pin1-c3.* An insertion of a T 858 bp downstream of the *PIN1* start codon resulted in a frameshift and deletion of the phosphorylation site S3 as well as the C-terminal part of the PIN1 protein. Note that because of sequencing quality, some bases on the left side were erroneously called. D) *pin1-c4.* An A is inserted 3091 bp downstream of the *PIN1* start codon. E) *pin1-c5.* A 51-bp fragment between the highlighted sequences in was deleted from the *PIN1* gene, resulting in the removal of 17 amino acid residues in the hydrophilic loop near transmembrane domain 6. The deleted peptide is GGNNISNKTTQAKVMPP. The left side of each deletion/insertion is highlighted in yellow, and the right side is highlighted in blue.

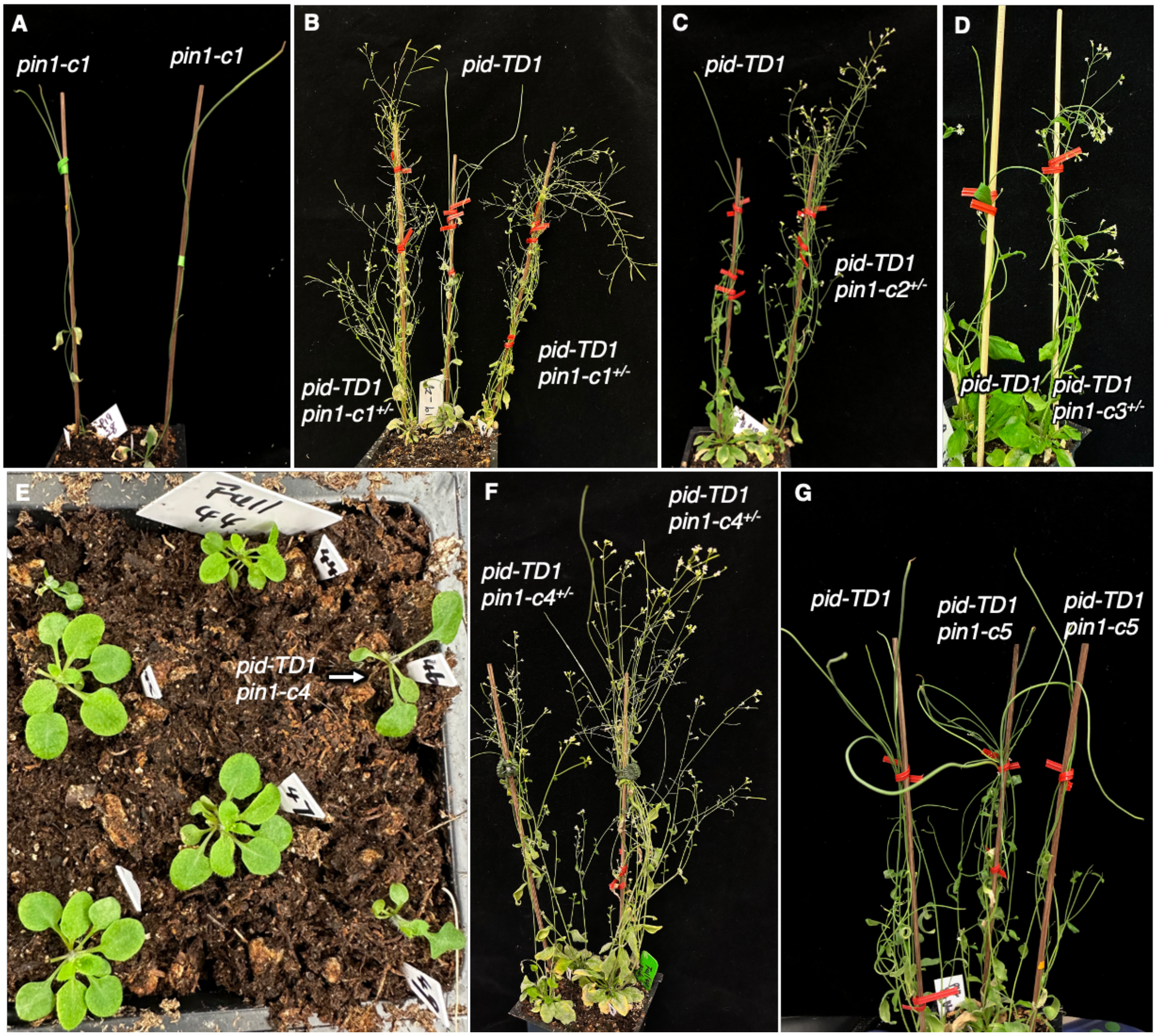

Fig. S5. Genetic interactions between various *pin1* mutations with *pid-TD1*. A) The *pin1-c1* allele is a strong allele of *pin1*, which makes pin-like inflorescences. B) Suppression of *pid-TD1* by heterozygous *pin1-c1*. Note the obvious flowers and siliques from *pid-TD1 pin1-c1+/-* plants whereas *pid-TD1* is completely sterile. C) Suppression of *pid-TD1* by heterozygous *pin1-c2*. D) Heterozygosity of *pin1-c3* suppresses *pid-TD1*. E) Homozygous *pin1-c4*, which is a weak allele, enhances *pid-TD1*. Note the pin inflorescence and fewer leaves (arrow). F) *pid-TD1* is suppressed by heterozygous *pin1-c4*. G) *pin1-c5* does not enhance or suppress *pid-TD1*.

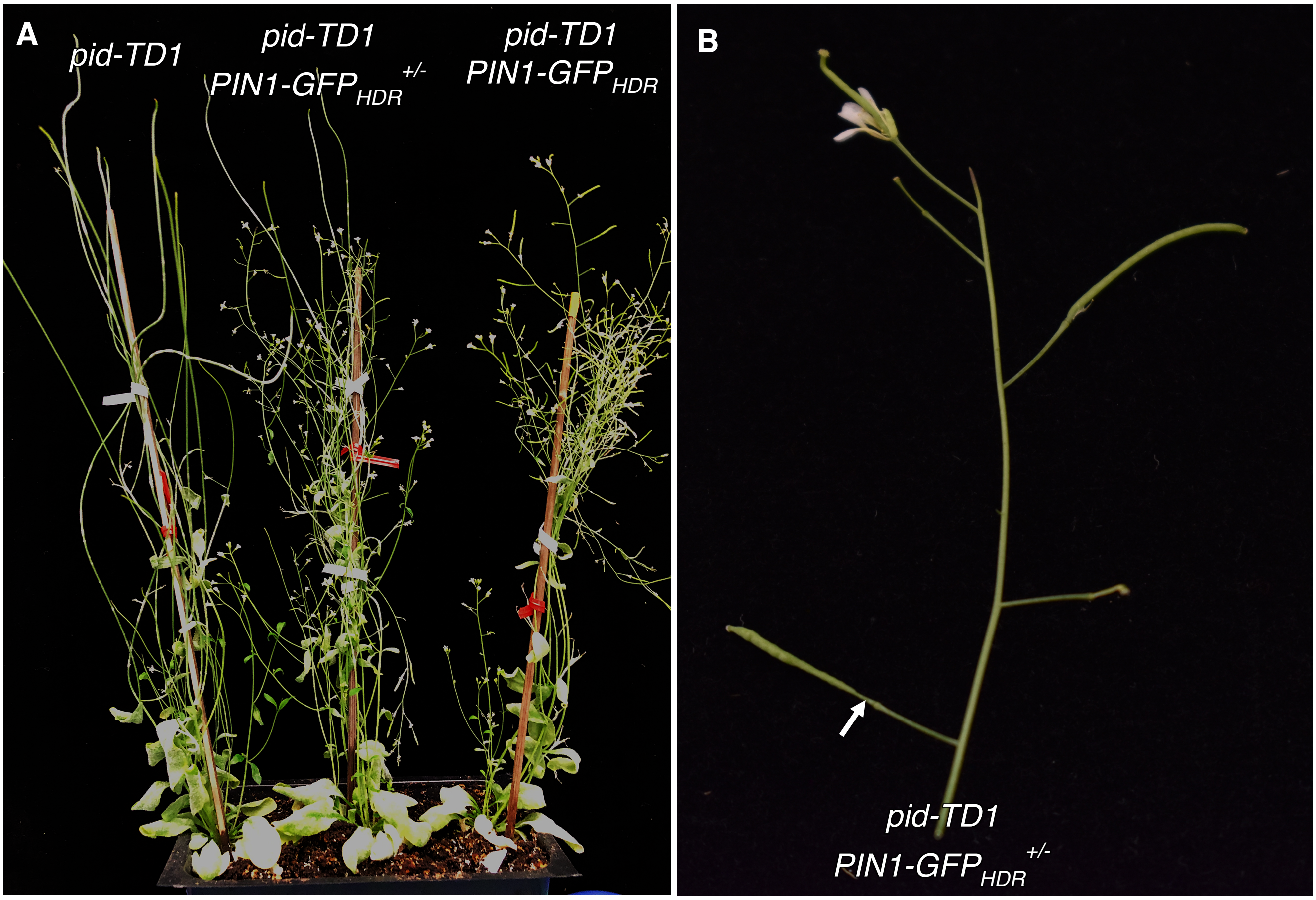

Fig. S6. Suppression of a *pid* null mutant by *PIN1-GFP_{HDR}* in a semi-dominant fashion. A) a single copy of *PIN1-GFP*$_{HDR}$ is sufficient to restore the fertility of *pid1-TD1.* Plant homozygous for *pid-TD1* (left) hardly make any flowers and produce pin-like inflorescences. The sterile pin-like phenotypes of *pid-TD1* are rescued by two copies of *PIN1-GFP*$_{HDR}$ (right). In the presence of just one copy of *PIN1-GFP*$_{HDR}$, *pid-TD1* is able to produce flowers and set viable seeds (middle). B) Heterozygous *PIN1-GFP*$_{HDR}$ is sufficient to restore fertility of *pid-TD1*. Note that not all flowers in *pid-TD1 PIN1-GFP*$_{HDR}$*/PIN1* are fertile and that the siliques sometimes have a long gynophore (arrow). *The pid-TD1*$^{+/-}$ *PIN1-GFP*$_{HDR}^{+/-}$ plants still make pin-like inflorescences.

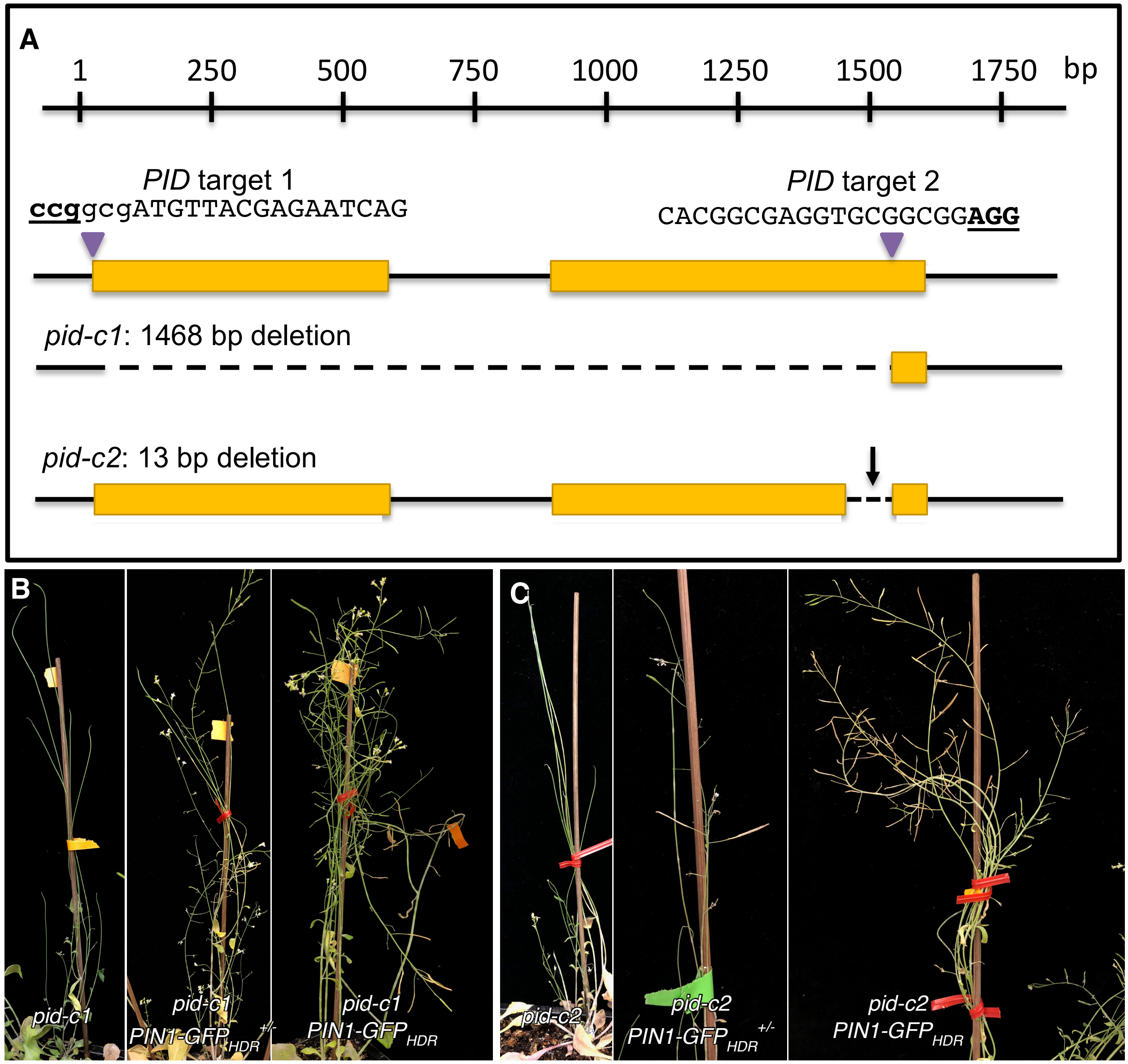

Fig. S7. Suppression of *pid* by *PIN1-GFP$_{HDR}$* is not allele dependent. A) Two new *pid* mutants were generated via CRISPR/Cas9 gene editing. The *pid-c1* allele has a large deletion which includes the loss of the start codon, and *pid-c2* has a 13 bp deletion which causes a frameshift in the second exon. Both alleles were generated using the two indicated gRNA target sequences. B) The strong allele *pid-c1* produces pin-like inflorescences. *PIN1-GFP$_{HDR}$* suppresses *pid-c1* in a semi-dominant fashion. C) One or two copies of *PIN1-GFP$_{HDR}$* also suppress the *pid-c2* phenotype.

```
                                                                S231
               β2      β3           S218               T227  (S1)
PIN1   IMSLDGRQPLETEAEIKEDGKLHVTVRRSNASRSDIYSRRS------QGLSATPRPSNLT   234
PIN2   VISLNGREPLQTDAEIGDDGKLHVVVRRSSAASSMISSFNKSHGGGLNSSMITPRASNLT   240
PIN3   VVSLDGHDFLETDAEIGDDGKLHVTVRKSNA------SRRSFCG-----PNMTPRPSNLT   229
PIN4   VVSLDGHDFLETDAEIGNDGKLHVTVRKSNA------SRR--------SLMMTPRPSNLT   226
PIN7   VVSLDGHDFLETDAQIGDDGKLHVTVRKSNA------SRRSFYGGG--GTNMTPRPSNLT   232
                  S252                  S271
              T248 (S2)                 (S4)
PIN1   NAEIYSLQSSRNPTPRGSSFNHTDFYSMMAS------GGGRNSNFG------PGE--AVF   280
PIN2   GVEIYSVQSSREPTPRASSFNQTDFYAMFNASKAPSPRHGYTNSYGGAGAGPGGDVYSLQ   300
PIN3   GAEIYSLST----TPRGSNFNHSDFYNMMGF------PGGRLSNFG------PADMYSVQ   273
PIN4   GAEIYSLSS----TPRGSNFNHSDFYSVMGF------PGGRLSNFG------PADLYSVQ   270
PIN7   GAEIYSLNT----TPRGSNFNHSDFYSMMGF------PGGRLSNFG------PADMYSVQ   276
            S290
 S282 T286 (S3)
PIN1   GSKGPTPRPSNYEEDGGPAKPTAAGTAAGAGRFHYQSGGS--GGGGGAHYPAPNPGMFSP   338
PIN2   SSKGVTPRTSNFDEEVMK-------TAKKAGRGGRSMSGELYNNNSVPSYPPPNPMFTG-   352
PIN3   SSRGPTPRPSNFEENC---------AMASSPRFGYYPGG---GAGS---YPAPNPEFSST   318
PIN4   SSRGPTPRPSNFEENN---------AVKYGFYNNTNSSVP--AAGS---YPAPNPEFSTG   316
PIN7   SSRGPTPRPSNFEESC---------AMASSPRFGYYPGG---APGS---YPAPNPEFS--   319

PIN1   ---NTGGGGGTAAKGNAP------VVGGKRQDGNGRDLHMFVWSSSASPVSDVFG---GG   386
PIN2   ---STSGASGVKKKESGGGGS----GGGVGVGGQNKEMNMFVWSSSASPVSEANA-----   400
PIN3   TTSTANKSVNKNPKDVNTNQQTTLPTGGKSNSHDAKELHMFVWSSNGSPVSDRAGLNVFG   378
PIN4   --TGVSTKPNKIPKENQQQLQ----EKDSKASHDAKELHMFVWSSSASPVSDVFG----G   366
PIN7   ---TGNKTGSKAPKENHH-------HVGKSNSNDAKELHMFVWGSNGSPVSDRAGLQVDN   369

PIN1   GGNHHADYSTATNDHQKDVKISVPQGNSN---------------------------DNQY   419
PIN2   ---KNAMTRGSSTDVSTDPKVSIPP-HDNLATKAMQNLIENMSPGR-----------KGH   445
PIN3   GAPDNDQGGRSDQG-AKEIRMLVPDQSHNGETKAVAHPASGDFGGEQQFSFAGKEEEAER   437
PIN4   GAGDNVATEQSEQG-AKEIRMVVSDQPRKSNARGGGDDIGGLDSGE---------GERE-   415
PIN7   GA--NEQVGKSDQGGAKEIRMLISDHTQNGENK--AGPMNGDYGGE---------EESER   416

          S426                                6a
PIN1   VEREEFSFGNKDDDSKVLATDGGNNISNKTTQAKVMPPTSVMTRLILIMVWRKLIRNPNS   479
PIN2   VEMDQDGNNG-GKSPYMGKKGSDVEDGGPGPRKQQMPPASVMTRLILIMVWRKLIRNPNT   504
PIN3   PKDAENGLNKLAPNSTAALQSKTGLGGAEASQRKNMPPASVMTRLILIMVWRKLIRNPNT   497
PIN4   IEKATAGLNKMGSNSTAELEAAGGDGGGNNG--THMPPTSVMTRLILIMVWRKLIRNPNT   473
PIN7   VKEVPNGLHKLRCNSTAELNPKEAIETGETVPVKHMPPASVMTRLILIMVWRKLIRNPNT   476
```

Fig. S8. Phosphorylation sites in PIN proteins. Protein sequences of the hydrophilic loops of the "long PINs" (PIN1,2,3,4,7) were aligned using ClustalW[1]. Alignments were manually altered to indicate the conserved "SRRS" motif starting at PIN1 residue S218. The seven phosphorylated residues identified from five *pid-TD1 PIN1-GFP* phospho-peptides are highlighted in yellow. Other conserved phosphorylation sites as reviewed previously (cite Barbosa 2018 paper) are bolded.

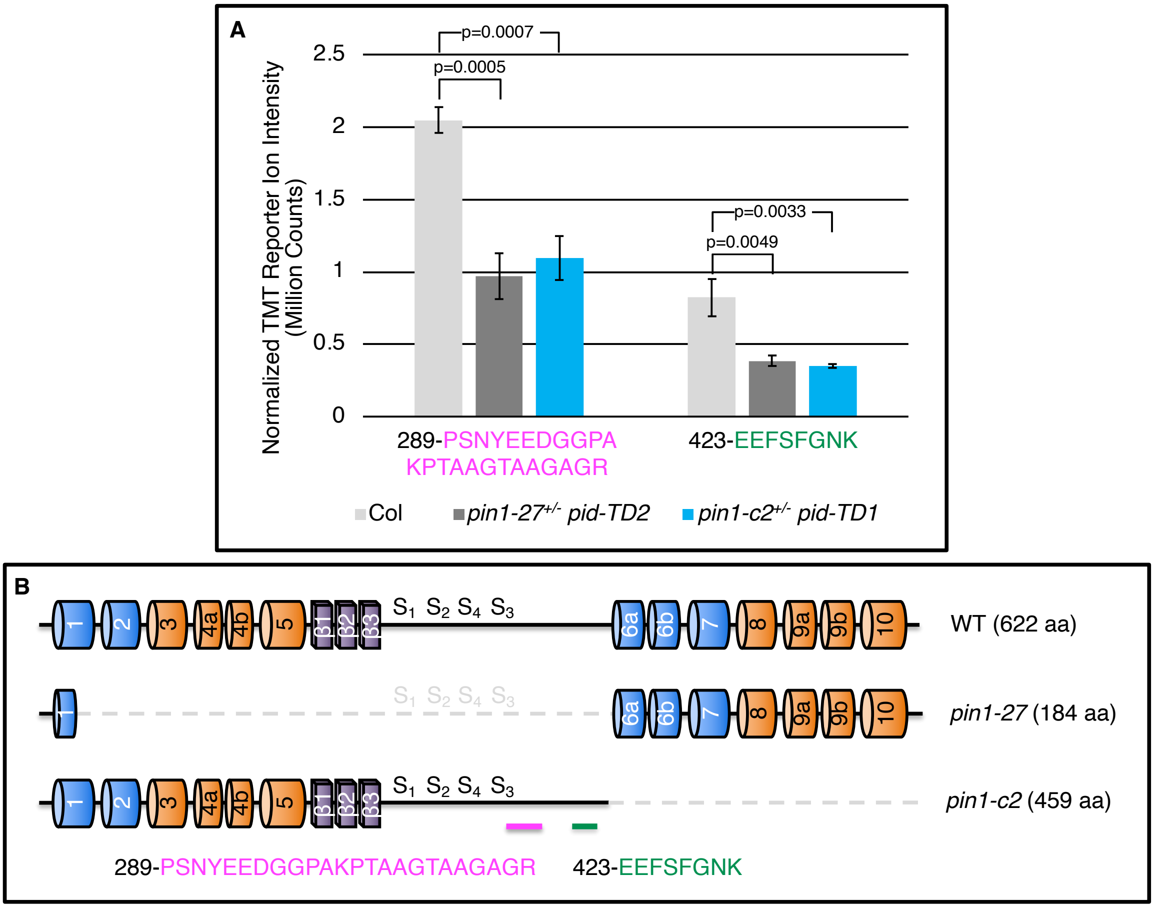

Fig. S9. Identification of PIN1 peptides in *pin1* mutants. A) Two PIN1 peptides were detected in mass spectrometry analysis of seedling tissues. B) The relative locations of the identified peptides and their presence or absence in the mutant *pin1* alleles. The two peptides are present in *pin1-c2* but not in *pin1-27*. Note that *pin1-27*$^{+/-}$ *pid-TD2 and pin1-c2*$^{+/-}$ *pid-TD2* refer to progenies from plants with those genotypes.

Table S1. CRISPR/Cas9 targets used to generate *pin1* and *pid* mutants

Primer Name	Primer Sequences	Purpose
S1-gRNA1	CCTTTGGAAACTGAAGCTGAGA	Together with S1- gRNA2, it can delete S1 site in PIN1. Combining with GIS-gRNA2, it can delete the hydrophilic loop of PIN1
S1-gRNA2	TCGTCGTTCTAATGCTTCAAGG	Delete S1 site in PIN1
S3-gRNA1	CCTACTCCGAGACCTTCCAACT	Delete S3 site in PIN1
S3-gRNA2	CCTTCCAACTACGAAGAAGACG	Delete S3 site in PIN1
GIS-gRNA1	AGTATTGGCAACGGACGGTGGG	Delete the site for GFP insertion
GIS-gRNA2	CCACCAACAAGTGTGATGACAA	Delete the site for GFP insertion
KO-gRNA1	CTTCTCTACTACATTCTCTTGG	KO-gRNA1 + KO-gRNA2 deletes PIN1 ORF
KO-gRNA2	TACCACGTTATGACGGCTATGG	KO-gRNA1 + KO-gRNA2 deletes PIN1 ORF
GFP-gRNA	AACAAAACGACGCAGGCTA**AGG**	Insert GFP in PIN1 through HDR
PID-gRNA1	CTGATTCTCGTAACATcgc**cgg**	PID-gRNA1 +PID-gRNA2, deletes PID
PID-gRNA2	**CCT**CCGCCGCACCTCGCCGTGA	PID-gRNA1 +PID-gRNA2, deletes PID

Table S2. Genotyping primers used in this study

Primer Name	Primer Sequences	Purpose
PIN1-SGT1	TCTTTGAGTACCGTGGAGCTAAGC	Genotyping pin1 CRISPR mutants
PIN1-SGT2	GCCACCACTTCCTCCAGATTGATA	Genotyping pin1 CRISPR mutants
PIN1-S-GT3	CAGGTGATGCCGAATAAACTGGA	Genotyping pin1 CRISPR mutants
PIN1-SGT4	ATCTTCACACCAGACCAATGCTCC	Genotyping pin1 CRISPR mutants
PIN1-sGT5	TCCACCGCTACGAACGATCATCA	Genotyping pin1 CRISPR mutants
PIN1-GFP-out1	GTCGGAACTCTAACTTTGGTCCT	PIN1-GFP-out1 + PIN1-GFP-out2 for determining zygosity of PIN1-GFP HDR
PIN1-GFP-out2	ATAAGCAGAGAACTGTGGAGCAT	PIN1-GFP-out1 + PIN1-GFP-out2 for determining zygosity of PIN1-GFP HDR
PIN1-GFP-IN-1	GACGGTGGGAACAACATAAGCA	With PIN1-GFP-out2, determines the presence of GFP insertion
PIN1-GFP-IN-2	GAGTCTTGTCATCACACTTGTTGG	with PIN1-GFP-out1, determines the presence of GFP insertion
PID-KO-RP2	cgaacgccgctggtttg	Genotype pid-TD mutants
PID-KO-LP2	gatgttacgagaatcagacg	Genotype pid-TD mutants
JMLB1	GGCAATCAGCTGTTGCCCGTCTCACT GGTG	Genotype pid-TD mutants
PID-NGT1	Gaagagagatcatgcaactggtcgg	Genotype pid-c mutant

Dataset S1 (separate file). Phospho-peptide data from mass spectrometry analysis of PIN1-GFP plants.

SI References

1. Higgins D., Thompson J., Gibson T. Thompson J. D., Higgins D. G., Gibson T. J. CLUSTAL W: improving the sensitivity of progressive multiple sequence alignment through sequence weighting, position-specific gap penalties and weight matrix choice. Nucleic Acids Res. 22:4673-4680. (1994)
